# Provincial Trends in Childhood Vaccine Coverage in Afghanistan From 2000 to 2024: An Ecological Study of Findings From the Institute for Health Metrics and Evaluation

**DOI:** 10.7759/cureus.109407

**Published:** 2026-05-21

**Authors:** Ahmad Khan, Melanie Tidman

**Affiliations:** 1 Health Sciences, A.T. Still University, Albuquerque, USA

**Keywords:** access, afghanistan, coverage, lower-income countries, vaccine

## Abstract

This study analyzes vaccination coverage trends across 34 provinces in Afghanistan from 2000 to 2024, using data from the Institute for Health Metrics and Evaluation (IHME). Coverage rates for bacillus Calmette-Guérin (BCG), diphtheria-tetanus-pertussis third dose (DTP3), measles-containing vaccine first dose (MCV1), polio vaccine (three doses), pneumococcal conjugate vaccine third dose (PCV3), hepatitis B vaccine third dose (HepB3), *Haemophilus influenzae* type b vaccine third dose (Hib3), and rotavirus vaccine (RotaC) were assessed using the Kruskal-Wallis and Friedman tests. The Friedman test and Kruskal-Wallis H test were used to assess significant differences in vaccination coverage trends across the 34 provinces in Afghanistan from 2000 to 2024, enabling a comprehensive analysis of provincial and temporal trends.

A downward trend in vaccination coverage was observed during the COVID-19 pandemic and after international aid limitations following the political change that occurred in Afghanistan in 2021. This decline in coverage may be associated with disruptions in vaccine distribution during these critical periods. Besides individual factors, vaccination adherence is influenced by health system factors, including vaccine availability and accessibility.

The results of the Kruskal-Wallis and Friedman tests showed significant variability in vaccination coverage trends across the 34 provinces from 2000 to 2024. These findings highlight the need for intensive efforts to improve vaccination coverage in all 34 provinces to protect those most at risk.

## Introduction

Vaccinations are a fundamental element in saving lives and reducing the burden of communicable diseases, death, and disability among populations [1]. Although vaccines have markedly reshaped global health by eradicating smallpox and reducing poliovirus incidence, vaccine coverage disparities continue to exist, especially in lower- and middle-income countries [2,3]. Afghanistan faces challenges in achieving widespread vaccination coverage due to limited funding, ongoing conflicts, inadequate health system infrastructure, and poverty [4,5].

Multiple immunization programs have been implemented in Afghanistan to improve health outcomes among vulnerable populations, particularly children under five, who represent the primary demographic [6]. Even with international aid, community participation, and government-led action, Afghanistan continues to face multiple challenges, such as vaccine hesitancy, security-related service disruptions, and difficulties in reaching remote provincial areas [7,8]. Variations in vaccine uptake across the country's 34 provinces, driven by these factors, have led to uneven coverage. Vaccines are vital catalysts for public health transformation and improvements in population well-being, helping to limit the spread of diseases such as measles, polio, and hepatitis, which may result in mortality and morbidity [9]. Extensive vaccination controls diseases, decreases the health burden, and improves workforce productivity [10].

Using data from the Institute for Health Metrics and Evaluation (IHME) provides a crucial opportunity to conduct a comprehensive analysis of vaccine coverage trends across 34 provinces from 2000 to 2024. The primary purpose of this research is to evaluate trends in the coverage of eight vaccines - bacillus Calmette-Guérin (BCG), diphtheria-tetanus-pertussis third dose (DTP3), measles-containing vaccine first dose (MCV1), polio vaccine (three doses), pneumococcal conjugate vaccine third dose (PCV3), hepatitis B vaccine third dose (HepB3), *Haemophilus influenzae* type b vaccine third dose (Hib3), and rotavirus vaccine (RotaC) - across Afghanistan’s provinces and over time from 2000 to 2024, a period marked by changes in Afghan political structures, the health sector, and international support and assistance. In Afghanistan, vaccine-preventable communicable diseases pose a threat to child health, quality of life, and survival. According to the World Health Organization, overall immunization coverage in Afghanistan remains low, with disparities across the country, particularly between rural and urban areas and between secure and insecure zones among children under five. The most vulnerable populations are those residing in hard-to-reach areas [11].

Mapping provincial disparities in vaccine coverage enables the Afghan health sector and international aid organizations to target interventions more effectively and allocate resources where they are needed most.

## Materials and methods

The primary objective of this retrospective ecological analysis is to assess vaccine coverage trends across the 34 provinces of Afghanistan over time, from 2000 to 2024, using vaccination data collected by the IHME (https://www.healthdata.org/research-analysis/health-topics/vaccine-coverage). The study investigates vaccinations in children less than one year old, including BCG, DTP3, MCV1, and polio vaccine (three doses). Additional vaccines included PCV3, HepB3, Hib3, and RotaC.

The mean vaccination coverage for each vaccine type over the specified years at the provincial level was used, and estimated infant populations were contextualized to estimate the proportion of young children who received the vaccines. The purpose of this study is to highlight areas of need and potential interventions to improve immunization coverage and health outcomes for infants and young children in Afghanistan.

For this ecological analysis, vaccine coverage data were obtained from the IHME Nextcloud folder, which provides comprehensive data for over 100 countries. A total of 850 available observations were downloaded specifically for Afghanistan, including vaccine coverage data across 34 provinces from 2000 to 2024 for DTP1, BCG, HepB3, Hib3, Polio3, RotaC, MCV1, PCV3, and DTP3. The data were then organized in IBM SPSS Statistics for Windows, Version 28 (released 2021; IBM Corp., Armonk, NY, USA), with variables arranged to assess coverage trends across the 34 provinces and temporal trends from 2000 to 2024. This structured approach ensures the replicability of the analysis conducted in this study.

The IHME has developed a vaccine coverage mapping tool that uses survey data, primarily from the Demographic and Health Surveys (DHS) and UNICEF Multiple Indicator Cluster Surveys (MICS), to provide local vaccination patterns. The vaccination status mapping data are based primarily on children’s vaccine cards, with parental recall used when cards are unavailable [12].

The production of vaccine coverage maps involves a multi-step mathematical modeling process that combines data from various sources to improve predictive accuracy across countries and years. Initially, a set of covariates, including population density, educational levels, and city access, is assembled from satellite imagery [13]. An ensemble model is then used to analyze these covariates and vaccine coverage data to make predictions in data-sparse areas. A geostatistical model is applied to correct for covariate inaccuracies, refine predictions, and identify local structural patterns [14,15]. Finally, the maps are calibrated using national vaccination data from the GBD study to ensure accuracy and reliability. Population estimates for children younger than one year are produced by WorldPop by blending country-specific, bottom-up data with top-down modeling and calibrating them to GBD national statistics [16].

The maps for BCG, DTP1, DTP3, MCV1, and Polio3 are regularly updated with new data and aim to reflect vaccine doses administered through routine immunization (RI). Polio3 coverage indicates a three-dose vaccine series in children who have received three doses of any polio vaccine (oral polio vaccine or inactivated polio vaccine) and does not count extra doses received during supplementary immunization activities (SIAs). DTP3 coverage is accurately estimated by directly modeling DTP1, while the DTP3-to-DTP1 ratio is also modeled to ensure that DTP1 coverage remains greater than or equal to DTP3 coverage, thereby enabling accurate DTP3 coverage calculations.

The estimates for PCV3, HepB3, Hib3, and RotaC vaccines are updated annually to incorporate new data. To produce subnational coverage estimates for these recently introduced vaccines, a spline-model approach is used to account for the non-linear scale-up of vaccine coverage shortly after introduction. This modeling uses the coverage of the established reference vaccine (DTP3 for PCV3, HepB3, Hib3, and RotaC; MCV1 for MCV2) as a benchmark, ensuring that newer vaccine estimates remain consistent with established vaccine performance. PCV3 and RotaC are modeled directly using DTP3 data, allowing the data to justify higher adoption rates. A three-stage modeling approach is implemented to address data sparsity, incorporating global trends into national and subnational estimates while maintaining flexibility in data-rich areas.

## Results

The results of this study provide a detailed examination of trends in vaccination coverage over a 24-year period across the 34 provinces of Afghanistan for the target population aged less than one year. With a sample size of 850 observations, the data were obtained from the IHME. IBM SPSS Statistics (IBM Corp.) was used for data analysis to assess the relationship between vaccination coverage trends from 2000 to 2024 using the Friedman test and Kendall’s Coefficient of Concordance (W) analyses. The Kruskal-Wallis test was used to assess statistically significant differences in vaccination coverage trends among the 34 provinces of Afghanistan. These non-parametric statistical methods were selected for their suitability in analyzing aggregated vaccination coverage data, specifically the mean values sourced from the IHME Nextcloud folder.

The Friedman test and the Kruskal-Wallis H test were used to assess the general trend in vaccination coverage across the 34 provinces over time from 2000 to 2024. While the absence of repeated-measures modeling may limit the ability to account for intra-provincial correlations and temporal trends, the ecological nature of this analysis highlights broader patterns in vaccination coverage. The results highlight disparities in vaccination coverage across the 34 provinces and provide valuable insights into the effectiveness of vaccination programs from 2000 to 2024.

The Kruskal-Wallis test was used to analyze differences in vaccination coverage for eight vaccines across the 34 provinces of Afghanistan. The results indicated that DTP1, BCG, Hepatitis B3, Hib3, Polio3, MCV1, and DTP3 showed significant variation (p < 0.001) across provinces, whereas PCV3 and rotavirus showed p-values of 0.754 and 1.000, respectively, indicating no significant differences (Table 1).

**Table 1:** Comparison of Vaccination Coverage Across 34 Provinces of Afghanistan Using the Kruskal-Wallis H Test This table presents the results of the Kruskal-Wallis H test assessing differences in vaccination coverage for bacillus Calmette-Guérin (BCG), the third dose of diphtheria-tetanus-pertussis (DTP3), the first dose of measles-containing vaccine (MCV1), and the three-dose polio vaccine (Polio3), the third dose of the pneumococcal conjugate vaccine (PCV3), the third dose of the hepatitis B vaccine (HepB3), the third dose of the *Haemophilus influenzae* type b vaccine (Hib3), and the rotavirus vaccine (RotaC) across the 34 provinces of Afghanistan. Kruskal-Wallis H values indicate the test statistic for each vaccine; (df) denotes degrees of freedom, and p-values indicate statistical significance. A p-value of < 0.001 indicates a statistically significant difference in coverage, whereas p-values of 0.754 and 1.000 indicate no significant difference in vaccine coverage for PCV3 and rotavirus, respectively. Source: [16].

Vaccine	DTP1	BCG	HepB3	Hib3	PCV3	Polio3	RotaC	MCV1	DTP3
Kruskal-Wallis H	216.15	310.046	125.392	91.381	27.136	195.815	5.74	402.614	190.674
df	33	33	33	33	33	33	33	33	33
p-value	< 0.001	< 0.001	< 0.001	< 0.001	0.754	< 0.001	1	< 0.001	< 0.001

The Friedman test was conducted to determine whether there were significant differences in vaccine coverage across the 34 provinces from 2000 to 2024. The results indicated a significant difference in the rankings of the different vaccines, as evidenced by a chi-square statistic of 5692.166 (p < 0.001). The BCG vaccine, 8.63 (21.61%), had the highest mean rank, indicating the highest coverage across the 34 provinces from 2000 to 2024 among the vaccines assessed. In contrast, the RotaC had the lowest mean rank, 1.68 (4.21%), indicating lower coverage than the other vaccines.

Additionally, Kendall’s Coefficient of Concordance (W) was calculated to evaluate the degree of agreement among vaccine coverage values, yielding a value of 0.837. This high value of Kendall’s W suggests a strong level of concordance in vaccine coverage, further reinforcing the significant differences identified in the Friedman test. A Bonferroni correction for multiple comparisons was used; the adjusted alpha level was set at approximately 0.005. All vaccines exhibited p-values below this adjusted threshold, indicating that the observed differences in vaccine coverage were statistically significant (Table 2).

**Table 2:** Ranks and Statistical Analysis of Vaccination Coverage in Afghanistan This table summarizes the vaccine ranks, coverage percentages, and statistical analysis results for various vaccines based on the Friedman test, with a sample size of 850 observations. The data were obtained from the Institute for Health Metrics and Evaluation (IHME) and represent vaccination coverage across different vaccines: first dose of diphtheria-tetanus-pertussis (DTP1), 7.49 (18.73%); bacillus Calmette-Guérin (BCG), 8.63 (21.61%); third dose of hepatitis B vaccine (HepB3), 3.49 (8.74%); third dose of *Haemophilus influenzae* type b vaccine (Hib3), 3.14 (7.85%); three-dose polio vaccine (Polio3), 7.21 (18.05%); rotavirus vaccine (RotaC), 1.68 (4.21%); first dose of measles-containing vaccine (MCV1), 5.80 (14.51%); third dose of pneumococcal conjugate vaccine (PCV3), 2.32 (5.80%); and third dose of diphtheria-tetanus-pertussis vaccine (DTP3), 5.22 (13.06%). Based on the Friedman test, the chi-square value of 5692.166 with degrees of freedom (df = 8) indicates significant differences in vaccine coverage from 2000 to 2024, as shown by p < 0.001. Furthermore, Kendall’s Coefficient of Concordance (W = 0.837), along with its associated chi-square value of 5692.166 and p < 0.001, indicates that variation in vaccine coverage from 2000 to 2024 was not random. A Bonferroni correction for multiple comparisons was applied; the adjusted alpha level was set at approximately 0.005. All vaccines exhibited p-values below this adjusted threshold, indicating that the observed differences in vaccine coverage were statistically significant. Source: [16].

Vaccines	Rank	p-value	Significant (Adjusted p < 0.00556)	Percentage (%)	Other Statistics
DTP1	7.49	<0.001	Yes	18.73	Friedman Test N: 850
BCG	8.63	<0.001	Yes	21.61	Chi-square: 5692.166
HepB3	3.49	<0.001	Yes	8.74	df: 8
Hib3	3.14	<0.001	Yes	7.85	Overall p-value: <0.001
Polio3	7.21	<0.001	Yes	18.05	Kendall’s Coefficient of Concordance N: 850
RotaC	1.68	<0.001	Yes	4.20	Kendall’s W: 0.837
MCV1	5.80	<0.001	Yes	14.51	Chi-square: 5692.166
PCV3	2.32	<0.001	Yes	5.80	df: 8
DPT3	5.22	<0.001	Yes	13.06	Overall p-value: <0.001

**Figure 1:**
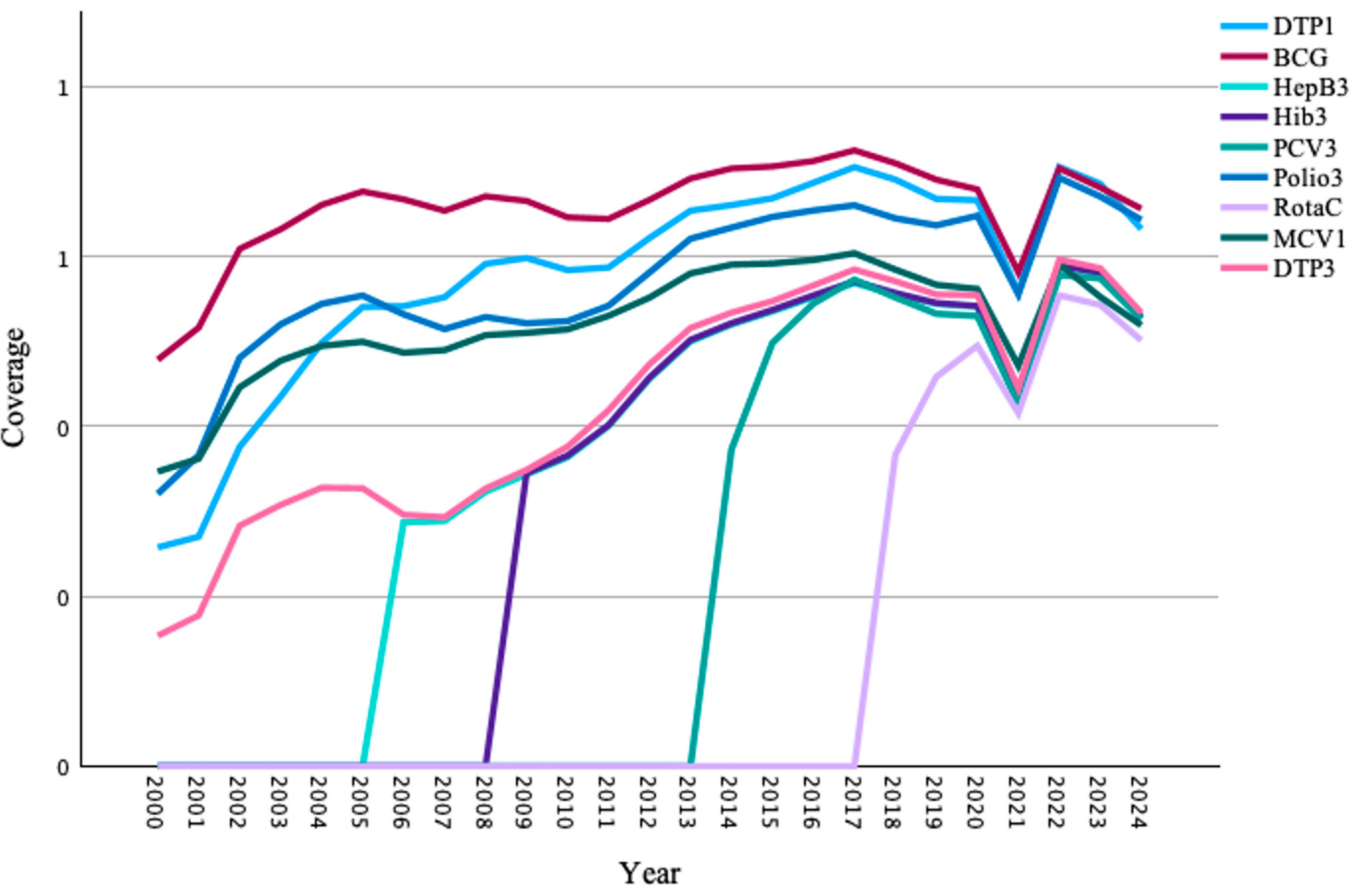
Vaccine Coverage Across 34 Provinces of Afghanistan: Trends From 2000 to 2024 This line graph presents a visual representation of vaccine coverage trends for bacillus Calmette-Guérin (BCG), the third dose of diphtheria-tetanus-pertussis (DTP3), the first dose of measles-containing vaccine (MCV1), and the three-dose polio vaccine (Polio3), the third dose of the pneumococcal conjugate vaccine (PCV3, introduced in 2013), the third dose of the hepatitis B vaccine (HepB3), the third dose of *Haemophilus influenzae *type b vaccine (Hib3), and the rotavirus vaccine (RotaC, introduced in 2017), across the 34 provinces of Afghanistan from 2000 to 2024. Source: [16].

Furthermore, a line graph was used to illustrate the spatial coverage of DTP1, BCG, HepB3, Hib3, Polio3, RotaC, MCV1, and PCV3 across the 34 provinces in Afghanistan from 2000 to 2024. The graph showed a decline in vaccine coverage during the COVID-19 pandemic. Following the initial pandemic phase in 2021, a continued downward trajectory in vaccine coverage was observed, indicating persistent challenges in maintaining immunization programs (Figure 1).

## Discussion

Vaccine access, availability challenges, and shortages in lower-income countries pose a threat to global public health, worsening the spread of preventable diseases among at-risk populations [17,18]. Analyzing provincial vaccine coverage trends over time is essential for identifying immunization gaps and optimizing vaccine distribution to reduce morbidity and mortality related to vaccine-preventable diseases [19,20]. This retrospective ecological analysis of provincial vaccine coverage trends across Afghanistan’s 34 provinces over time, from 2000 to 2024, uses comprehensive datasets collected by the IHME. Comparing multi-year and provincial data in Afghanistan highlights vaccine availability and shortages.

Vaccination coverage, plotted on a line graph across the 34 provinces from 2000 to 2024, declined during the COVID-19 pandemic and after the political change, with international aid curtailed after 2021, warranting further investigation. Similar patterns were observed in other lower-income countries such as Yemen, Pakistan, and Bangladesh, as well as higher-income countries such as Sweden, the United States, and Norway, where RI plummeted during the COVID-19 pandemic [21-24]. Globally, coverage declined between 2019 and 2021, reaching its lowest point since 2015; for example, DTP1 decreased from 90% to 86%, and DTP3, MCV1, and Polio3 decreased from 86% to 81% [25]. According to the World Health Organization, international aid reductions resulted in vaccination coverage dropping from approximately 70% in 2019 to 37% in 2021 for critical vaccines in Afghanistan [26]. These findings are consistent with other countries, such as a systematic review of empirical evidence from 1985 to 2025, which indicated that armed conflicts and political instability were associated with declines in childhood vaccination coverage [27].

Furthermore, Kruskal-Wallis and Friedman analyses show considerable variations in vaccination coverage across vaccine types from 2000 to 2024, with differences observed across the 34 provinces, aligning with trends observed in other low-income economies [28]. The DTP3 and BCG vaccines had statistically significant differences in coverage across the provinces (Kruskal-Wallis H = 190.674 and 310.046, both p < 0.001). The Friedman test analysis of vaccine coverage indicates greater coverage for the BCG vaccine, with a coverage rank of 8.63 (21.61%), and DTP1, 7.49 (18.73%), compared to the RotaC, 1.68 (4.21%), and PCV3, 2.32 (5.80%). These trends are consistent with observations reported in other low-income countries [28]. This discrepancy may be related to suboptimal public health campaigns and resource allocation toward established versus newer vaccines [29].

In Afghanistan, the low uptake of newer vaccines can be due to limited public awareness and limited access, as similar patterns are seen in other lower-income countries [30]. These gaps have widened during the COVID-19 pandemic and with a drop in international aid. Research has shown that funding from global health initiatives significantly boosts vaccination coverage in lower-income regions [31]. According to UNICEF in Afghanistan, vaccination interruptions have led to a spike in polio cases due to political instability, a weakened health system, and population movement, highlighting the urgent need for effective vaccination programs [32]. 

The Essential Program on Immunization has fundamentally altered the course of global health, contributing to children’s survival, facilitating the elimination of diseases, and expanding public health infrastructure [33]. Future research in this area needs to identify potential factors driving variation in vaccination across Afghanistan’s 34 provinces to improve vaccination coverage among vulnerable populations.

Limitations of the study

In rural parts of Afghanistan over a two-decade period, accurate tracking of vaccine coverage is limited by the possible lack of written immunization records, leading to reliance on verbal reports from parents. Recall bias poses a high risk of misreporting true immunization coverage. Moreover, regional differences in data collection methods in Afghanistan, including variation in vaccination card formats and availability, may lead to discrepancies in reported data. In the last two decades, wars and conflicts in some parts have increased security, logistical, and transportation challenges that raise concerns over the accurate assessment of vaccine coverage. Targeting only the population under one year old can pose a limitation, as this age range does not account for the timing of catch-up vaccinations. Using a low-resolution 5 × 5 km² mapping approach may overlook considerable disparities in vaccination coverage in urban areas. This approach may fail to capture meaningful differences between adjacent socioeconomically contrasting neighborhoods. Furthermore, confounding factors may affect the accuracy of vaccine coverage estimates across the 34 provinces, such as parents’ socioeconomic status, level of education, and cultural beliefs regarding childhood immunization. Future research is needed to consider these confounding factors to enhance the reliability of findings. Finally, the study’s methodological limitations are due to its ecological nature, which lacks individual-level data.

## Conclusions

To summarize, vaccination coverage in Afghanistan from 2000 to 2024 showed considerable variability across the nine vaccines assessed in this study and across the 34 provinces. Variation in vaccination coverage may stem from several factors at the individual, health system, public health communication, and logistical levels, which warrant further investigation. Future research should focus on identifying the root causes of these disparities to inform area-based interventions across all provinces of Afghanistan.
